# The impact of an exercise physiologist coordinated resistance exercise program on the physical function of people receiving hemodialysis: a stepped wedge randomised control study

**DOI:** 10.1186/1471-2369-14-204

**Published:** 2013-09-27

**Authors:** Paul N Bennett, Robin M Daly, Steve F Fraser, Terry Haines, Robert Barnard, Cherene Ockerby, Bridie Kent

**Affiliations:** 1 Centre for Nursing Research - Deakin University and Monash Health Partnership, Clayton, Victoria 3168, Australia; 2 Centre for Physical Activity and Nutrition Research, School of Exercise and Nutrition Sciences, Deakin University, Burwood, Victoria 3125, Australia; 3 Allied Health Research Unit, Kingston Centre, Monash University - Monash Health, Cheltenham, Victoria 3192, Australia; 4 Centre for Physical Activity in Ageing, Northfield, South Australia 5085, Australia; 5 School of Nursing and Midwifery, Plymouth University, Plymouth, Devon PL4 8AA, UK

**Keywords:** Exercise, Resistance training, Hemodialysis, Dialysis, Stepped wedge, Cluster randomised control trial, Cost utility analysis, Exercise physiologist

## Abstract

**Background:**

Exercise during hemodialysis treatments improves physical function, markers of cardiovascular disease and quality of life. However, exercise programs are not a part of standard therapy in the vast majority of hemodialysis clinics internationally. Hemodialysis unit-based accredited exercise physiologists may contribute to an increased intradialytic exercise uptake and improved physical function.

**Methods and design:**

This is a stepped wedge cluster randomised controlled trial design. A total of 180 participants will be recruited from 15 community satellite hemodialysis clinics in a large metropolitan Australian city. Each clinic will represent a cluster unit. The stepped wedge design will consist of three groups each containing five randomly allocated cluster units, allocated to either 12, 24 or 36 weeks of the intervention. The intervention will consist of an accredited exercise physiologist-coordinated program consisting of six lower body resistance exercises using resistance elastic bands and tubing. The resistance exercises will include leg abduction, plantar flexion, dorsi flexion, straight-leg/bent-knee raise, knee extension and knee flexion. The resistance training will incorporate the principle of progressive overload and completed in a seated position during the first hour of hemodialysis treatment. The primary outcome measure is objective physical function measured by the 30-second sit to stand test. Secondary outcome measures include the 8-foot timed-up-and-go test, the four square step test, quality of life, cost-utility analysis, uptake and involvement in community activity, self-reported falls, fall’s confidence, medication use, blood pressure and morbidity (hospital admissions).

**Discussion:**

The results of this study are expected to determine the efficacy of an accredited exercise physiologist supervised resistance training on the physical function of people receiving hemodialysis and the cost-utility of exercise physiologists in hemodialysis centres. This may contribute to intradialytic exercise as standard therapy using an exercise physiologist workforce model.

**Trial registration:**

Current Controlled Trials ACTRN12612001223820.

## Background

End stage kidney disease leads to accelerated physical deterioration, decreased mobility, decreased capacity for independence and an increased risk of falling [1,2]. Previous research indicates that 40% of people receiving hemodialysis treatment have a significant falls-related injury each year, with 54% of these resulting in fractures [3]. A recent Cochrane Review confirmed that exercising at least three times per week for greater than 30 minutes per session for people with end stage kidney disease was effective for improving physical fitness, walking capacity, blood pressure and health-related quality of life [4]. Despite this, there are very few hemodialysis centres that provide intradialytic exercise programs as standard therapy [5,6]. A major contributing factor to this is the lack of professional exercise expertise of the hemodialysis nurses and technicians in the hemodialysis clinics [7]. In Australia, accredited exercise physiologists (AEPs) are masters level university trained allied health professionals who specialize in prescribing safe and effective exercise programs to clinical populations at high risk of developing, or with one or more, chronic diseases [8]. A recent study reported that without the introduction of qualified exercise professionals, exercise programs in hemodialysis clinics are unlikely to be sustainable [9].

To our knowledge, no research study using a stepped wedge randomised controlled design has been undertaken to examine the effect of an exercise physiologist-coordinated resistance exercise program for people attending hemodialysis clinics. Although our previous pilot studies suggest that exercise physiologists can increase exercise participation in hemodialysis clinics [10,11], the small sample size and short duration of these studies limited the conclusions that could be drawn. Thus a larger more rigorous trial is required to confirm the pilot data.

The primary objective of this research is to measure the effect of an AEP-coordinated exercise program on physical function determined by the 30-second sit to stand test. Secondary outcome measures include the 8-foot timed-up-and-go test, the four square step test, quality of life, cost-utility analysis, uptake and involvement in community activity, self-reported falls, fall’s confidence, medication use, blood pressure and morbidity (hospital admissions).

It is hypothesised that an AEP-coordinated exercise program will improve the physical function of people with end stage kidney disease who are receiving hemodialysis. This work is expected to be significant given the effects of end stage kidney disease on musculoskeletal health and the high number of severe falls in this group. The current absence of exercise programs in outpatient hemodialysis clinics (commonly known as satellite hemodialysis clinics) means there are few sustained exercise programs in Australia or internationally, leading to a decline in hemodialysis patients’ physical function and quality of life, and increased costs to health services.

## Methods

### Study design and setting

This study will use a stepped wedge cluster randomised controlled trial design. This method is a form of randomised controlled trial where randomisation occurs while still allowing all participants the opportunity to undertake a period of intervention [12]. In this study, the randomisation will occur at a clinic level rather than individual participant level. Participants will be recruited from 15 community satellite hemodialysis clinics across five healthcare organisations in Melbourne, Australia. Each clinic will represent a cluster unit. The stepped wedge design will consist of three groups each containing five randomly allocated cluster units. The first group will receive 36 weeks of the AEP-coordinated exercise intervention, the second group will receive 24 weeks, and the third group will receive 12 weeks. The study will be conducted over a 48 week period, incorporating a 12 week pre-intervention period. The study design was reviewed by the Australian New Zealand Clinical Trials Registry (ANZCTR), approved and registered as ACTRN12612001223820 on the 19th November, 2012.

### Ethical considerations

The study has been approved by all five intervention health network ethics committees and the Chief Investigator’s university ethics committee: Southern Health Human Research Ethics Committee (#12376B), Deakin University Human Research Ethics Committee (#2013-024), Eastern Health Human Research Ethics Committee (#E43-1213), Fresenius Nephrocare Medical Affairs Department (#4313), Austin Health Research Ethics Unit (#H2013/04990) and Alfred Hospital Ethics Committee (#87/13).

Participants will be asked to sign two separate consent forms; one will document their consent to participate in the intervention and evaluation (general consent) and the second will document their specific consent for the researchers to request data from Medicare Australia about medical services accessed and medications prescribed for the duration of the study (Medicare consent). All participants will be asked to sign both consent forms, but they may still be recruited into the study if they decline to sign the second form. This means that they will participate in all other aspects of the intervention and evaluation but their Medicare data will not be accessed.

### Recruitment of participants

Flyers advertising the study will be placed on patient noticeboards in each of the clinics from which participants will be recruited for this trial. All patients who are interested in participating will be advised to contact their nurse clinic manager or the research team directly. Once they have registered their interest, a research assistant completely independent of the hemodialysis clinic will speak with the patient in person at a subsequent hemodialysis treatment. The research assistant will provide a verbal explanation of the study and also a copy of the Participant Information and Consent Form. The patients will be advised to read the information and encouraged to take it home to relatives and significant others to discuss if preferred. If they choose to participate they will return their completed consent form to a secured box in the hemodialysis clinic.

### Inclusion and exclusion criteria

**Figure 1:**
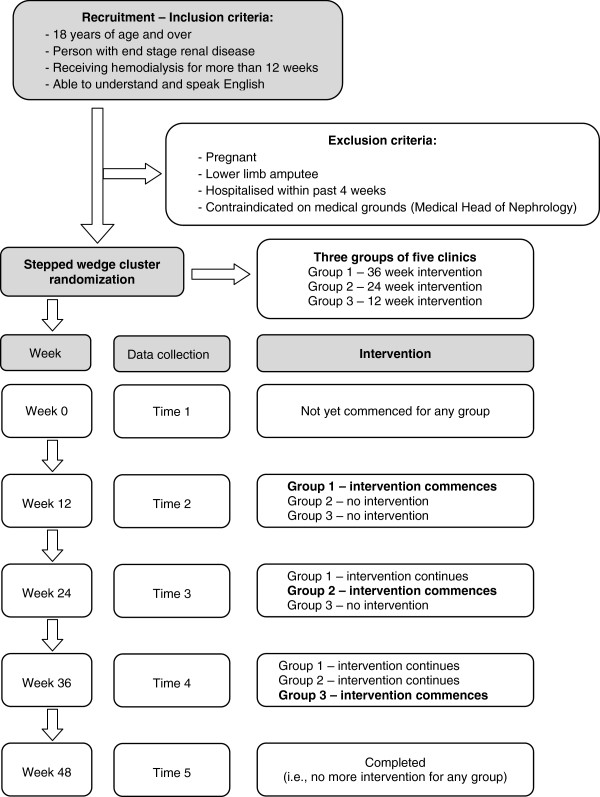
Flow chart of trial.

Participants will be included in the study if they have end stage renal disease and are receiving hemodialysis, are aged 18 years and over, able to understand and speak English and on hemodialysis greater than 12 weeks (Figure 1). People will be excluded if they are pregnant, have had lower limb amputation or have been hospitalised in the four weeks prior to study commencement. Additionally, the medical Head of Nephrology at each organisation will identify any patient they consider not suitable on medical grounds for the intervention.

### Randomization

An individual not associated with the trial will perform the randomization using the Microsoft Office Excel™ computer-generated random number system. Each cluster (each dialysis clinic) will be randomly prescribed a number and the five clinics with the 5 lowest numbers will be designated into Group 1 (receiving 36 weeks of the exercise intervention), numbers 6–10 will be allocated to Group 2 (receiving 24 weeks of the exercise intervention) and the final 5 clinics will be allocated to Group 3 (receiving 12 weeks of the exercise intervention). Allocation will be concealed at the time of participant consent; however the participants and clinic staff will be made aware of their group at the commencement of the intervention. Blinding of intervention group number to clinical staff and dialysis patient participants will not be possible once the intervention commences.

### Intervention

Accredited exercise physiologists (AEPs) will be appointed to each hemodialysis clinic six hours per week per clinic for the duration of the intervention. Depending on which group the hemodialysis clinics are randomised to, participants will receive an exercise program that is implemented for 12, 24 or 36 weeks. Each participant will be assessed by an AEP who will develop an individualised exercise program [10]. Consistent with our previous pilot study, a typical program will consist of six lower body resistance exercises using elastic bands and tubing. The exercise program will be done during the first hour of hemodialysis treatment. When participants are able to perform two sets of 15–20 repetitions for each exercise, the resistance exercises will be made progressively harder using different colour-graded elastic bands and tubing. Participants will also be encouraged to perform each exercise (lifting phase - concentric contraction) as rapidly as possible to optimise movement speed and muscle power. The resistance exercises to be incorporated into the program include: leg abduction, plantar flexion, dorsi flexion, straight-leg/bent-knee raise, knee extension and knee flexion. All resistance exercises will be done in a seated position so the person maintains a comfortable position while still receiving hemodialysis treatment. The AEP will conduct the program once per week for each participant over the intervention period. Participants will also be encouraged to perform the exercises during the two hemodialysis treatments where the AEP is not present. The AEP will provide training about the correct technique and progressions for each exercise to nurses in the hemodialysis clinic so that they can monitor patients on days when they are not present. Adherence rates will be recorded by the nursing staff using a tick box on the patient’s daily hemodialysis treatment record and daily exercise cards completed by the participants, both of which will be reviewed weekly by the AEP.

### Data collection

Baseline data (Time 1) for all participants will be collected 12 weeks prior to the commencement of the intervention for the first group. The second round of data collection (Time 2) will be conducted 12 weeks later and participants from the five clinics randomised to receive 36 weeks of intervention (Group 1) will commence the exercise intervention. The third round of data collection (Time 3) will be conducted 12 weeks later and participants from the five clinics randomised to receive 24 weeks of intervention (Group 2) will commence the exercise intervention. The fourth round of data collection (Time 4) will be conducted 12 weeks later and participants from the five clinics randomised to receive 12 weeks of intervention (Group 3) will commence the exercise intervention. The final round of data collection will be conducted in the week after the intervention ceases in all clinics, 48 weeks after the first data is collected (Time 5) (Table 1).

**Table 1:** Outcome measures

**Data collected at 0, 12, 24, and 36 and 48 weeks**	
Physical function	• 30 second sit and stand test
	• 8 foot up and go
	• 4 square step test
Involvement in community activities	• Frenchay activities index • Dialysis exercise adequacy
Falls	• Boston falls calendar
	• Modified falls efficacy scale
Biochemistry and dialysis treatment	• Midweek pre-dialysis serum phosphate, serum calcium, potassium, urea, creatinine, albumin, urea reduction ratio
	• Midweek pre-dialysis blood pressure
	• Post-dialysis weight
Cost utility analysis	• Hospital admissions: Number, reason for admission, length of stay
	• Medications
	• Kidney dialysis quality of life – short form
	• Utilisation of non-Medicare health services (e.g., physiotherapy, district nursing)
	• Pharmaceutical benefits scheme^*^
	• Medical benefits schedule^*^

^*^Data collected only at 48 weeks.

### Primary outcome measure

Objective physical function is the primary outcome measure in this study and will be measured using the 30-second sit to stand test. The test will follow the protocols developed by Rikli and Jones [13] and Dite and Temple [14], and is a simple, non-invasive test that can be conducted with minimal equipment in the hemodialysis clinic. The 30-second sit to stand test has been validated for use with older people and people with chronic disease [13,14]. Tests will be conducted before participant’s midweek hemodialysis treatment every 12 weeks from the start date by an AEP who is blinded to the intervention group.

### Secondary measures

Secondary outcome measures will include two further physical function tests (the eight foot timed up and go test and the four square step test), quality of life, community activity involvement, dialysis exercise adequacy, falls and falls confidence, medication use, biochemical measures, morbidity and cost. Quality of life will be measured at every 12 weeks from study commencement using the validated Kidney Dialysis and Quality of Life Index (KDQOL) [15]. KDQOL reliability and validity have been confirmed in the hemodialysis population [15]. Uptake and involvement in community activities such as shopping, social outings, and gardening will be measured using the Frenchay Activities Index (FAI) every 12 weeks from study commencement. The FAI has been validated in people with chronic disease [16]. Dialysis Exercise Adequacy (DEA) will be measured every 12 weeks from study commencement using the formula: weekly exercise frequency (number of times) × time (hrs) × age/100 [17]. This will include exercise both within and outside of the intervention and will identify any change in the uptake of overall exercise over the course of the study. Self-reported falls will be measured every 12 weeks from study commencement by asking each participant if they had any falls in the past 12 weeks, how many falls they had over four weekly periods, and whether an injury was sustained, based on the falls reporting method used in the Mobilize Boston Study [18]. In this study, a fall will be defined as “an event, which results in a person coming to rest inadvertently on the ground or other lower level” [19]. In addition, falls confidence will be measured every 12 weeks from study commencement using the Modified Falls Efficacy Scale [20]. Participant use of medication in terms of the number and type of medications will be extracted from medication charts. This information will be collected at the commencement of the intervention for each group (Time 2, 3 or 4), and at the completion of the study (Time 5). Biochemical and dialysis-related data routinely measured during hemodialysis (i.e., mid-week post-dialysis weight, mid-week pre and post sitting blood pressure) will be recorded every 12 weeks from study commencement. Morbidity will be measured by number of hospital admissions, reason for hospital admission and length of stay and will be recorded for all participants for the duration of the study. This information will be obtained verbally from the participant every 12 weeks from study commencement. If clarification is required (i.e., participant is unable to recall details), further information may be sought from the participants’ hemodialysis treatment progress record that is kept in the hemodialysis clinic. Medication and morbidity data will contribute to cost utility analysis. Unit costs will be derived from data obtained from Medicare Australia regarding Medicare Benefits Schedule (MBS) and Pharmaceutical Benefits Scheme (PBS) claims (i.e., net cost, rebate, gap payment) for each participant for the study duration, and from Australian Refined Diagnosis Related Groups (AR-DRG) cost weights [21]. Participants will also be asked if they have accessed any health services that are not recorded via Medicare (e.g., physiotherapy, district nursing) every 12 weeks, to more accurately estimate their health-related costs. Quality adjusted life years (QALYs) effect will be estimated using the SF-6D algorithm for KDQOL data [22] and an Incremental Cost Effective Ratio (ICER) will be calculated [23].

### Study flow

The study flow is detailed in Figure 1. Firstly clinics will be randomized to group 1, 2 or 3. After participants are screened for eligibility based on the inclusion/exclusion criteria and provide written informed consent, they will undergo baseline physical function tests 12 weeks prior to the commencement of the intervention. At the completion of these tests, the investigator will explain and ask the participant to take home the combined questionnaire which will include the KDQOL, FAI, Boston Falls Calender and the modified falls efficacy scale. Participants will be asked to complete these questionnaires and return them at their next dialysis visit. Data obtained will be transcribed onto case record forms for entry into a specifically designed trial database. Data analysis will then be undertaken by an investigator who is blinded to the group intervention. The data collection plan is detailed in Table 1.

### Study withdrawal

Participants can withdraw from the intervention at any stage, effective immediately, without affecting clinical care as documented and explained at time of consent. Participants who withdraw will be invited to consent to follow-up testing for the remainder of the trial to enable an intention-to-treat data analysis.

### Sample size calculation

The 30 second sit-to-stand test is the most clinically meaningful primary outcome measure and was used in the sample size calculations for this study. It is clinically meaningful because many people with chronic kidney disease on hemodialysis have great difficulty simply getting out of bed or out of a chair. This inability to stand quickly from a chair is associated with their poor muscle condition [24,25] and renal bone disease [26]. Likely effect sizes have been estimated from the sit-to-stand test data from two previous studies [27,28]. In Cappy et al. [27] the effect sizes (d) for the three, six, and twelve-month changes in reps were 0.65, 1.12, and 2.39, respectively. In McDonald et al. [28] the effect size (d) for the 12-week change was 2.42. We chose the most conservative findings with the lowest effect size (Cappy’s 3-month change: d = 0.65) for the sample size calculations. Cappy et al. [27] demonstrated a medium-sized effect for the effect of a physical activity intervention (d = 0.65), alpha was set at p < 0.0125 (an adjustment was made to the typical level of p < 0.05 for judging statistical significance in these types of studies, because there will be a test at each of four time points and we wish to control experiment wise error). Power was set at .80. Based on our previous pilot work a 30% attrition rate over 12 months is expected [10]. A design effect (DE = 1.25) was included to account for clustering (ICC = .029, m = 9.73). The intra-cluster correlation coefficient was estimated from prior work with people with diabetes in primary care practices: ICC = .029 [29]. We will require 15 clusters, with each cluster representing one hemodialysis clinic. Based on these assumptions, 180 participants will need to be recruited for the study. Thus in each of the 15 clinics we will need to recruit a minimum of 12 participants.

### Statistical analyses

For the primary outcome measure there will be five data collection points, one every 12 weeks as follows: Time 1 (0 weeks, no intervention), Time 2 (12 weeks, Group 1 commences intervention), Time 3 (24 weeks, Group 2 commences intervention), Time 4 (36 weeks, Group 3 commences intervention) and Time 5 (48 weeks, intervention completed). This design characteristic means analyses will occur at three time points (Time 3 to Time 5) with the data at each of these time points compared with baseline data. Three tests means that the standard α value (p < 0.05) will need to be divided by three (a Bonferroni adjustment): α = .05/3 = .0167. Initially, analyses of variance with baseline scores as co-variates (ANCOVAs) will be the method of analysis. In addition, to account for the clustering effect, generalised linear mixed models with random effects will be used to analyse differences between control period and intervention period.

## Discussion and conclusion

The primary outcome for this study is to examine the effect of an AEP-coordinated resistance exercise intervention on hemodialysis patients’ physical function. A secondary associated outcome is the cost utility of exercise physiologists together with community activity involvement, dialysis exercise adequacy, falls and falls confidence, medication use, biochemical measures and morbidity. The importance of these measures is significant given the paucity of uptake of exercise in hemodialysis clinics worldwide. Exercise physiologists have increasingly been involved in the improved outcomes of people with chronic conditions; however, this has not been tested in the hemodialysis population. This study will provide an improved understanding on the impact of an AEP-co-ordinated resistance exercise intervention and whether it is a cost effective model for health services providing hemodialysis.

## Competing interests

The authors declare that they have no competing interests.

## Authors’ contributions

PNB, RMD, SFF, RB, BK, CO and TH are responsible for the design of this trial, and the construction of the protocol. TH provided advice on cost utility analysis. All authors read and approved the final manuscript.

## Pre-publication history

The pre-publication history for this paper can be accessed here:

http://www.biomedcentral.com/1471-2369/14/204/prepub
